# Efficacy of Panobinostat for the Treatment of Multiple Myeloma

**DOI:** 10.1155/2020/7131802

**Published:** 2020-01-13

**Authors:** Evangelos Eleutherakis-Papaiakovou, Nikolaos Kanellias, Efstathios Kastritis, Maria Gavriatopoulou, Evangelos Terpos, Meletios Athanasios Dimopoulos

**Affiliations:** Department of Clinical Therapeutics, National and Kapodistrian University of Athens, School of Medicine, Alexandra General Hospital, Athens, Greece

## Abstract

Panobinostat represents a potent oral nonselective pan-histone deacetylase inhibitor (HDAC) with activity in myeloma patients. It has been approved by the FDA and EMA in combination with bortezomib and dexamethasone for the treatment of multiple myeloma, in patients who have received at least two prior regimens, including bortezomib and an immunomodulatory agent. In order to further explore its clinical potential, it is evaluated in different combinations in relapsed/refractory and newly diagnosed multiple myeloma. This review focuses on available data about panobinostat's pharmacology and its role in clinical practice. This review will reveal panobinostat's efficacy as antimyeloma treatment, describing drug evolution from preclinical experimental administration to administration in phase III trials, which established its role in current clinical practice. Based on the latest data, we will present its mechanism of action, its efficacy, and most important issues regarding its toxicity profile. We will further try to shed light on its role in current and future therapeutic landscape of myeloma patients. Panobinostat retains its role in therapy of multiple myeloma because of its manageable toxicity profile and its efficacy, mainly in heavily pretreated multiple myeloma patients. These characteristics make it valuable also for novel regimens in combination with second-generation proteasome inhibitors, IMiDs, and monoclonal antibodies. Results of ongoing trials are expected to shed light on drug introduction in different therapeutic combinations or even at an earlier level of disease course.

## 1. Introduction

Multiple myeloma is a plasma cell dyscrasia characterized by clonal plasma cell proliferation within bone marrow and increased production of monoclonal paraprotein, excreted in the blood or urine. It mainly affects elderly population, with a median age of diagnosis at approximately 70 years [1]. It is the third most common hematopoietic malignancy (after lymphoma and leukemia), representing approximately 13% of hematologic malignancies and 1% of all cancers [2, 3]. In 2018, it was estimated that 30,770 patients in the USA would be diagnosed with multiple myeloma and 12,770 patients will succumb to myeloma disease [4]. Globally, it is estimated that in 2018, 159,985 patients will be diagnosed with multiple myeloma and 106,105 patients will expire due to myeloma disease [5]. Due to continuous population aging, the incidence of myeloma is expected to rise in time. Typical clinical disease manifestations include anemia, hypercalcemia, renal insufficiency, and myeloma bone disease, known also as the “CRAB” features. Despite advances in disease's early detection, including recently introduced biological markers (abnormal FLC ratio, bone marrow infiltration by clonal plasma cells >60%, and more than one focal lesion in MRI), the aforementioned CRAB features remain the hallmark of active multiple myeloma disease [6].

Initial therapeutic management of multiple myeloma with conventional chemotherapy attained poor results [7, 8]. The introduction of novel agents [9], such as proteasome inhibitors and immunomodulatory drugs [10–13], and incorporation of autologous stem cell transplantation in clinical practice [14–16] has significantly reformed therapeutic landscape of multiple myeloma patients and vastly increased their outcome, by improving significantly the response rate and depth of response. Superior therapeutic efficacy of novel agents has been translated into prolonged progression-free survival (PFS) and overall survival (OS). Recent introduction of second-generation novel agents (such as carfilzomib [17] and pomalidomide [18, 19]) and monoclonal antibodies (such as daratumumab [20–24], isatuximab [25–28], and elotuzumab [29–31]) in multiple myeloma therapeutic setting has rapidly evolved therapeutic management, especially for refractory/relapsed multiple myeloma patients. Before the introduction of more advanced novel agents (carfilzomib and pomalidomide), patients with relapsed/refractory myeloma after initial therapy with proteasome inhibitors and IMiDs attained a dismal prognosis, with a median PFS of 5 months and a median OS not exceeding 9 months [32].

Despite major therapeutic advances in multiple myeloma therapy, it remains an incurable disease. Initial response to the aforementioned therapeutic agents is usually transient. Due to the evolvement of multiple malignant clones, multiple myeloma patients finally relapse, with the emergence of a more resistant myeloma cell population, requiring new lines of treatment. Most patients receive multiple lines of therapy during the course of their disease [33]. However, after each relapse, duration of subsequent response usually shortens, revealing an unmet medical need for effective therapies for heavily pretreated patients [34, 35]. The aforementioned data underline the importance of continuous research for agents with new mechanisms of action that will continue to offer a clinical benefit in multiple myeloma patients refractory/relapsed to current therapeutic regimens. Ideally, agents should be active through novel mechanisms of action and should be effective as monotherapy with panobinostat should resensitize patients to previously administered therapeutic agents.

Panobinostat (chemical name: 2-hydroxypropanoic acid, compound with 2-(E)-N-hydroxy-3-[4-[[[2-(2-methyl-1H-indol-3- yl)ethyl]amino]methyl]phenyl]-2-propenamide [1 : 1], trademark Farydak) is a first-in-class potent pan-deacetylase (DAC) inhibitor [36] that has been approved in February 2015 by the US FDA (US Food and Drug Administration) in combination with bortezomib and dexamethasone for the treatment of multiple myeloma, in patients who have received at least two prior regimens, including bortezomib and an immunomodulatory agent [37, 38]. The EMA (European Medicines Agency) has also granted approval in August 2015 for this agent [39], which is also recommended by the NICE (National Institute for Health and Care Excellence) [40] for the same indication.

## 2. HDAC System

DAC enzymes, also known as histone deacetylases (HDACs) are proteins that mainly act on histone proteins and transcription factors. Histones are responsible for DNA arrangement into nucleosomes. Histone modifications by histone deacetylases lead to different chromatin conformation, permitting DNA transcription [41]. Therefore, histone deacetylases and histone acetyltransferases by their opposing activities regulate gene expression, cell differentiation, and survival [42–45]. Some members of the HDAC family are transferred from the nucleus to cytoplasm and mainly act on nonhistone proteins. These proteins are categorized into four main classes (I, II, III, and IV). Preclinical studies with myeloma cells have shown that tumor suppressor genes are silenced in these cells [46, 47]. Treatment of myeloma cell lines with HDAC inhibitors like panobinostat induces reactivation of suppressed genes, leading eventually to cell death. In further studies with myeloma cells, DAC inhibition resulted in upregulation of genes that have been related to increased overall survival (OS) [48]. These preclinical trials demonstrated that pharmaceutical manipulation of HDACs had antimyeloma activity and should be further accessed in clinical trials. In fact, several DAC agents have been already evaluated in clinical trials like panobinostat, vorinostat [49–51], ricolinostat [52, 53], romidepsin [54], and ACY-241 [55]. Among HDAC inhibitors, panobinostat remains the only approved agent for the treatment of refractory/relapsed multiple myeloma patients.

## 3. Drug Chemistry

Panobinostat (LBH-589) is an orally administered hydroxamic acid pan-DAC inhibitor (Table 1) [36]. It has shown preclinical activity in all four classes of HDACs. At low concentrations, it induces apoptosis in multiple myeloma cells resistant to conventional therapies. Moreover, it inhibits cell proliferation even in the presence of BMSCs and it induces caspase activation and poly-(ADP-ribose) polymerase (PARP) cleavage in myeloma cell lines. It is worth to mention that deacetylase HDAC6 seems to regulate aggresome formation [56], an alternative cleavage pathway for catabolism of misfolded proteins that develop when proteasomes are incapable of cleaving misfolded ubiquitinated proteins. Therefore, proteasome inhibitors such as bortezomib, by inhibiting proteasome cleavage mechanism, induce aggresome formation due to overload of excess proteins while panobinostat through inhibition of HDAC6 blocks aggresome formation [57]. Their combination in myeloma cells lines results in synergistic toxicity against myeloma due to the complementary blocking of these complementary protein degradation pathways, the aggresome and the proteasome, leading to increased accumulation of excess proteins, and eventually signals the cell to undergo apoptosis. Bortezomib also targets and downregulates class I HDACs, indicating a different mechanism of HDAC inhibition, complementary to that of HDAC inhibitors [58–60]. These preclinical observations provided the rational for subsequent clinical trials combining panobinostat with bortezomib.

## 4. Pharmacokinetics and Metabolism

After receiving orally a dose of 20 mg panobinostat, it is rapidly absorbed, achieving maximum plasma concentration within 0.5 to 2 hours [61, 62]. When orally administered, the median maximum plasma concentration of panobinostat was 5.5–21.2 ng/ml. The absolute bioability of orally administered panobinostat was 21%. Drug bioability and rate of absorption was not significantly affected by food intake. Panobinostat has a half-life of 31 hours and is excreted in feces and urine, nearly equally among all patients [63, 64]. Panobinostat is metabolized via hydrolysis, acidosis, and glucuronidation through mainly noncytochrome P450 (CYP) enzymes and CYP enzymes. CYP3A4 inhibitors may significantly increase panobinostat exposure, thus requiring close monitoring for drug interactions or even panobinostat dose reduction. On the contrary, CYP34 inducers may significantly reduce panobinostat exposure and should be avoided when possible.

Panobinostat may also inhibit CYPY2D6 and relevant substrates should be avoided when possible. Panobinostat administration has been reported to induce QT prolongation, especially in intravenous administered form. Therefore, concomitant administration of agents that prolong QT should be avoided. Regarding panobinostat administration and renal or hematological impairment, earlier studies have shown that renal function did not affect drug clearance and tolerability while hepatic impairment was associated with reduced drug clearance and increased plasma exposure [65, 66].

## 5. Administration Schedule

According to manufacturer's labeling, panobinostat is orally administered in a schedule of 20 mg starting dose once every other day for 3 doses each week during weeks 1 and 2 of a 21-day cycle for up to 8 cycles [67]. The total duration of therapy may continue up to 16 3-week cycles if the patient shows clinical benefit and manageable toxicity. After eight cycles of treatment, bortezomib administration is modified while panobinostat dosing remains unchanged. Renal impairment does not require dose modification. In mild hepatic impairment, due to increased drug exposure, it is recommended to reduce panobinostat starting dose. Panobinostat administration is contradicted in severe hepatic impairment. If dose reduction is required, administration may be reduced at 5 mg intervals. Panobinostat should be discontinued if a dose of 10 mg three times a week is not tolerable.

## 6. Clinical Trials of Panobinostat in Multiple Myeloma Patients

### 6.1. Panobinostat as Monotherapy

Encouraging results from preclinical trials led to evaluation of monotherapy with panobinostat in clinical studies. In a pioneer phase Ia/II trial with various hematologic malignancies, maximum tolerated dose has been determined at 60 mg weekly [68]. In this trial, 12 patients with multiple myeloma received panobinostat and only one of them achieved PR (partial response), indicating a poor result as monotherapy. Panobinostat was further evaluated as monotherapy in a clinical trial with patients suffering from refractory/relapsed multiple myeloma. In a phase II trial, 38 heavily pretreated multiple myeloma patients (with a median of five prior therapies) received 20 mg panobinostat three times weekly until disease progression or intolerance [69]. Monotherapy with panobinostat resulted in also limited clinical activity with 2/38 patients achieving partial response and minor response (MR), respectively, which were durable. Furthermore, 9/38 patients achieved stable disease (SD).

### 6.2. Panobinostat with Bortezomib and Dexamethasone

Initial preclinical studies with myeloma cell lines indicating synergistic activity of panobinostat and bortezomib and poor results from clinical trials evaluating panobinostat monotherapy provided the rationale for clinical evaluation of combinations of panobinostat with bortezomib and dexamethasone. In a phase Ib trial establishing maximum tolerated doses (MTD) for panobinostat, bortezomib, and dexamethasone in refractory or relapsed and refractory multiple myeloma patients [70], 52.9% of patients achieved at least PR. It is worth to mention that among bortezomib refractory patients, 26.3% achieved at least PR, after reexposure to bortezomib with panobinostat, indicating that addition of panobinostat restored sensitiveness in a subgroup of previously refractory patients. These encouraging results led to further exploration of panobinostat's safety and effectiveness in a phase II trial (Panorama 2) [71] and in a phase 3 trial (Panorama 1) [72], in refractory/relapsed multiple myeloma patients.

Panorama 2 clinical trial included 55 patients. These patients had received at least 2 lines of treatment (with a median number of four previous lines of therapy, including an immunomodulatory drug) and were relapsed/refractory to bortezomib (progression within 60 days from the last dose of bortezomib). 34.5% of patients achieved at least PR with a median progression-free survival (PFS) of 5.4 months. This trial demonstrated the clinical benefit from panobinostat administration providing the rational for conducting a large multicenter randomized phase III trial, Panorama 1 trial. In this pivotal trial, 768 patients were randomly assigned in a 1 : 1 ratio to receive therapy with panobinostat-bortezomib-dexamethasone or placebo-bortezomib-dexamethasone. Patients had received 1 to 3 prior lines of treatment. Primary refractory myeloma patients and bortezomib refractory myeloma patients were excluded. The primary endpoint of this trial was PFS and was met as patients in the panobinostat arm achieved a median PFS of 12 months, compared to 8.1 months in the placebo arm (*p* < 0.0001). Median duration of response was also higher in the panobinostat arm (12 months vs. 7 months, respectively). Despite that, response rates were similar in both groups, patients receiving panobinostat achieved more frequently deeper than PR response (VGPR/CR) (28% vs. 16% compared to patients that received placebo (*p*=0.00006). There was a trend towards superior survival for patients that received panobinostat that was sustained in the final overall survival analysis of this trial but did not reach statistical significance [73]. Based on these favorable results, the FDA granted approval in February 2015 for panobinostat combined with bortezomib and dexamethasone for relapsed and refractory multiple myeloma patients who have received at least 2 prior lines of treatment, including bortezomib and an IMiD.

Panobinostat has been further evaluated in combination with bortezomib and dexamethasone as induction therapy in multiple myeloma patients eligible for autologous stem cell transplantation with high-dose melphalan as conditioning regimen [74]. Compared to clinically established bortezomib-based induction regimens (VRD, VCD, VTD), this combination attained lower response rate, lower quality of response at a cost of significant discontinuation rate, and frequent drug dose reduction, rendering it rather unsuitable as pretransplant regimen.

Noteworthy, in the aforementioned trials, bortezomib was administered mainly intravenously. In the PANEX clinical trial [75], 87% (34 of 39) of patients received subcutaneously bortezomib with panobinostat and dexamethasone. All patients were heavily pretreated with a median of four previously administered treatment regimens. In the subcutaneously administered bortezomib population, 62% of patients achieved at least PR and 15% of patients achieved minimal response. The combination demonstrated a marked clinical benefit with concerns about regimen tolerability, since 28% of patients discontinued therapy due to adverse events. Nonetheless, it is worth to notice the short duration of therapy (8.1 weeks), mainly due to progressive disease, as probably expected in this heavily pretreated group of patients.

### 6.3. Panobinostat and Carfilzomib

Synergistic activity of panobinostat and the first-in-class proteasome inhibitor bortezomib provided the rational for exploring potential synergy of panobinostat with more advanced proteasome inhibitors like carfilzomib. Preclinical data in human multiple myeloma cell lines where panobinostat and carfilzomib were combined indicated a synergistic inhibition of myeloma cell survival, by inducing apoptosis, mitochondrial injury, and caspase activation. Oxidative damage through generation of ROS (reactive oxygen species) and inhibition of ERK1/2 pathway that normally protects myeloma cells from apoptosis were considered to be the main mechanisms of carfilzomib-panobinostat-induced myeloma cells' apoptosis [76]. Based on initial encouraging preclinical data, a phase I/II study of carfilzomib/panobinostat in refractory/relapsed multiple myeloma patients has been conducted [77]. In this clinical trial, 44 heavily pretreated patients (median number of five prior regimens) were treated with this combination. Among them, 67% achieved at least PR irrespective of prior treatments. Adverse cytogenetics did not appear to affect response rate. The median time to progression (TTP) was 7.7 months while better quality of response predicted longer progression-free survival. After a median follow-up of 17 months, the overall survival has not been reached rendering this combination an effective regimen in these heavily pretreated patients. Combination of panobinostat with carfilzomib was further explored in another phase I trial [78]. 32 patients with a median number of 4 previously administered therapies received a median of 8 treatment cycles with carfilzomib and panobinostat. 57% of patients achieved at least PR with a median PFS of 8 months and a median OS of 23 months. Noteworthy, in both previously mentioned clinical trials, steroids were not administered, without affecting the efficacy and safety of these steroid-sparing regimens, which were equally effective in bortezomib-sensitive and bortezomib-refractory patients.

### 6.4. Panobinostat and Lenalidomide

Combination of lenalidomide and panobinostat was evaluated in a phase 2 clinical trial [79]. 27 heavily pretreated patients with a median of 3 prior lines of treatment received this combination. Among them, 17 (63%) showed high-risk cytogenetics. 41% of patients achieved at least PR, a remarkable result considering that 81% of patients were initially lenalidomide refractory. The median PFS was 7.1 months while in the lenalidomide refractory group, the median PFS was 6.5 months.

### 6.5. Panobinostat and Other Combinations

In vivo and in vitro preclinical data demonstrated synergistic activity of panobinostat with chemotherapeutic agents [80] like melphalan and anthracyclines [81]. Based on the aforementioned important data, a phase 2 study was designed, administering panobinostat combined with melphalan, thalidomide, and dexamethasone in relapsed/refractory multiple myeloma patients. Patients received 6 cycles of MPT-panobinostat and maintenance therapy with panobinostat plus prednisone. Due to severe drug-induced toxicity, dosage of panobinostat was reduced to 10 mg three times weekly. At least 45% of patients achieved at least PR with a median PFS at 8.1 months and a median OS at 18.2 months, at cost of significant toxicity [82].

Positive results from clinical trials combining bortezomib or thalidomide with panobinostat encouraged the evaluation of a VTD-panobinostat regimen for relapsed/refractory multiple myeloma patients [83]. A phase I/II open-label trial was conducted including 57 patients with refractory or relapsed multiple myeloma that had received a median of 1 line of treatment. Patients could receive 6 cycles of intense therapy and continue with panobinostat monotherapy as maintenance. 46 patients received the recommended dose of 20 mg for panobinostat. Among them, 91% achieved at least PR, with a median PFS of 16.1 months, and median OS was not reached, after a median follow-up of 28 months. Treatment was well tolerated. Most adverse events were mild or moderate with few cases of grade 3-4 diarrhea or fatigue, partially attributed to subcutaneous administration of bortezomib. In this trial, the VTD-panobinostat regimen emerged as an efficient and safe therapeutic modality. However, it must be underlined that 80% of patients had received only one previous line of treatment and patient refractory to bortezomib were excluded, leading to a highly selected drug-sensitive trial population. Favorable results in the first relapse prompted evaluation of panobinostat with VRD in a pretransplant phase I clinical trial, in order to potentially increase response rate and quality of response and reduce tumor burden before stem cell collection [84]. All 55 treated patients received at least one cycle of therapy. 95% of them achieved at least PR, and 22% of them achieved at least CR, a CR rate rather equal to previously reported for VRD regimen. Noteworthy, all patients who underwent stem cell collection after 4 cycles of VRD-panobinostat managed to collect a sufficient number of stem cells. VRD‐ panobinostat regimen has also been evaluated in refractory/relapsed multiple myeloma patients. 16 patients that had already received a median of four prior lines of therapy received this regimen achieving 5 months of PFS and 13 months of overall survival [85]. Finally, panobinostat has been evaluated in even more heavily pretreated patients [86], refractory after>10 previous lines of treatment, combined with carfilzomib, thalidomide, cyclophosphamide, and dexamethasone, offering disease control with manageable toxicity, justifying further evaluation of panobinostat-based combinational regimens.

### 6.6. Panobinostat and Immunotherapy

There are few available data regarding combination of panobinostat and immunotherapy in multiple myeloma. Enhanced antimyeloma activity of daratumumab and panobinostat combination has been demonstrated in preclinical models [87]. Panobinostat has been shown to increase CD38 expression on myeloma cells from patients with newly diagnosed multiple myeloma or refractory/relapsed disease. Increased CD38 expression has been associated with a higher response rate in daratumumab monotherapy, probably due to increased daratumumab-associated ADCC [88]. In order to exploit a possible synergistic activity of these agents, myeloma cell lines were subsequently exposed to panobinostat and daratumumab, eliminating much more myeloma cells compared to daratumumab monotherapy. Clinical trials are warranted to confirm if this combination may offer additional clinical benefit by increasing response rate and extending duration of response, compared to daratumumab monotherapy.

PD-1/PD-L1 axis has also been evaluated in myeloma cell lines with conflicting results. Immune checkpoint inhibitors have been evaluated in multiple myeloma with lenalidomide or pomalidomide with modest results, mainly due to increased toxicity, high risk of mortality, and uncontrolled immunoreactivity [89–92]. Ongoing trials are now exploring the combination of anti-PD-1/PD-L1 agents with anti-CD38 monoclonal antibodies. Combinations of HDAC inhibitors with anti-PD-L1 agents have been shown to synergistically reduce melanoma cell survival, due to HDAC inhibitors' ability to induce prolonged PD-L1 expression in both human and mouse melanoma cell lines [93, 94]. These combinations have also been evaluated in clinical trials (NCT02935790 and NCT02032810) in patients with advanced melanoma. Regarding multiple myeloma, preclinical studies have evaluated the combination of an anti-PD-L1 antibody with panobinostat [95] or other HDAC inhibitors, showing enhanced cytotoxicity [96]. These results should also be evaluated in clinical trials in order to confirm if restored immune function and enhanced cytotoxicity from these combinations may be translated in additional clinical benefit.

## 7. Safety Issues and Strategies of Management of Key Adverse Events

Panobinostat toxicities have been reported to be primarily of gastrointestinal and hematologic origin. Its toxicity profile shares strong resemblance to that of other HDAC inhibitors, including vorinostat and romidepsin. Initial trials with panobinostat monotherapy revealed main issues of drug's toxicity profile. Panorama I clinical trial provided a vast amount of data regarding panobinostat toxicity profile and its effective management. Most common severe adverse events (grade 3/4) were hematologic, including thrombocytopenia, anemia, and neutropenia [97, 98]. Gastrointestinal disorders and their management remain an important clinical issue for this group of patients. Diarrhea is the most common nonhematologic adverse event, affecting 76% of patients (33% of grade 3/4), followed by fatigue (60%, 26% of grade 3/4). Drug toxicities necessitated dose modification or even drug interruption in the vast majority of patients [72, 99–102]. Despite drug toxicities, the proportion of on treatment deaths was similar between panobinostat and placebo arm. Moreover, deaths due to progressive disease were reported to be being slightly higher in the placebo arm. Noteworthy, administration of early intravenous formulations of panobinostat showed the potential to prolong QT interval in some patients [103]. In subsequent clinical trials with the oral form, QT prolongation frequency was equal to placebo arm [104]. However, QT prolongation is a dose dependent drug-associated toxicity and can appear more frequently with increased doses of oral panobinostat. Moreover, arrhythmias and cardiac-related deaths were more frequently reported during panobinostat administration [71]. Therefore, patients should have an ECG with normal QTc interval before initiating therapy with panobinostat and have monitoring with repeated ECGs of QTc while on therapy. Concomitant medications known to prolong QT interval should be avoided if possible. Panobinostat is also not recommended in patients with a recent history of myocardial infarction.

Panobinostat toxicity profile necessitated the development of strategies for alleviating drug-induced toxicity. Hematologic toxicities are commonly described since most of the data are extracted from studies where panobinostat is combined to bortezomib and some of them may also be attributed to bortezomib-induced hematologic toxicity. Thrombocytopenia is a frequently reported adverse event (AE) in panobinostat regimens. It is attributed to the inhibition of megakaryocytes and the reduced release of proplatelets. Usually, delay or reduction of panobinostat dose is sufficient for management of thrombocytopenia. Appearance of diarrhea usually requires drug interruption, while in more severe forms (grade 3 or 4), drug dose reduction or even discontinuation may be necessary. Diarrhea can be a debilitating adverse event that may result in dehydration and electrolyte imbalance. Appropriate hydration, restoration of electrolytes, and prompt initiation of appropriate antidiarrheal medication are usually required. Regular electrolyte monitoring and prophylactic antiemetic therapy should also be considered.

Besides bortezomib, as already reported, panobinostat has been combined with other classes of antimyeloma agents. Many common treatment-emergent AEs may be attributed to the overlapping toxicity profiles of the various classes of agents involved. Despite the relatively limited information regarding toxicity profile of the combination of carfilzomib and panobinostat, safety profile of carfilzomib-panobinostat combination, particularly regarding thrombocytopenia, neurotoxicity, fatigue, and gastrointestinal disorders, compares favorably with that of panobinostat and bortezomib [77, 78]. Carfilzomib-panobinostat regimen necessitated less-frequent dose reductions due to toxicity, compared to established bortezomib-panobinostat-dexamethasone regimen. Combination of lenalidomide with panobinostat was also well tolerated with less commonly severe thrombocytopenia, fatigue, and diarrhea, compared to bortezomib, panobinostat, and dexamethasone standard regimen [79]. Regarding patients that received more complex combinations like VRD-panobinostat regimen [84, 85], they developed less frequently severe gastrointestinal toxic effects, at a cost of more often severe hematologic adverse events, particularly thrombocytopenia. In these patients, most frequent hematological grades 1-2 event was anemia and grade 3 event was thrombocytopenia, respectively. Most-frequent grade 3 nonhematological adverse events were fatigue and diarrhea. Despite that 45% of patients underwent dose reduction, no patient necessitated panobinostat's dose interruption.

## 8. Current Therapeutic Landscape in Multiple Myeloma and Future Directions

HDAC inhibitors represent a novel class of therapeutic agents with a unique mechanism of action. Panobinostat effectiveness as a part of combinatorial regimens granted its approval in the third line of treatment combined with bortezomib and dexamethasone. However, since panobinostat's approval, therapeutic armamentarium for efficient management of refractory/relapsed multiple myeloma patients has tremendously evolved. Second-generation novel agents like carfilzomib and pomalidomide, anti-CD38 monoclonal antibodies, and anti-SLAM7 monoclonal antibodies have proven therapeutic efficacy and have been incorporated in current clinical practice, forming the current landscape for multiple myeloma treatment.

In order to increase therapeutic efficacy, combinations of novel agents like monoclonal antibodies with IMiDs and proteasome inhibitors have been widely explored. These combinations attained deeper responses with prolonged disease control and manageable toxicity profile. Unfortunately, despite recent advances in myeloma management, almost all of patients will eventually relapse, highlighting the unmet medical need for agents with novel mechanisms of actions that will remain active in relapsed/refractory multiple myeloma patients or will resensitize myeloma patients to previously administered therapies.

Panobinostat is an effective drug with a predictable and manageable toxicity profile. Gastrointestinal toxicity in the form of diarrhea, hematologic toxicity, and rare cardiac events in the form of arrhythmias due to QT prolongation should be always taken under concern. Tolerability of this agent could be further increased by careful, proactive adverse event management. Despite reported toxicity, targeting HDAC remains an effective therapeutic option in selected subgroups of refractory/relapsed multiple myeloma patients. Panobinostat has demonstrated in phase II panorama 2 trial [71] the potential to resensitize heavily pretreated patients, refractory to bortezomib, achieving an overall response rate of 34.5% and a clinical benefit rate of 52.7%.

Moreover, ongoing clinical trials (Panorama 3) have been designed in order to investigate the safety and efficacy of three different regimens of panobinostat (20 mg TIW, 20 mg BIW, and 10 mg TIW) in combination with bortezomib in subcutaneous form and dexamethasone. This trial is going to identify if reduction in the dosing schedule of panobinostat and subcutaneous administration of bortezomib (which is the current standard procedure) may offer an effective regimen with more tolerable toxicity. Besides bortezomib, panobinostat has demonstrated in initial phase I/II trials a remarkable synergistic activity with carfilzomib and IMiDs, offering clinical benefit in heavily pretreated, refractory patients. Noteworthy, carfilzomib monotherapy in heavily pretreated (at least 4-5 previous lines of treatment) patients is reported to be far less efficient [105, 106] than combined with panobinostat, highlighting a potent clinical synergy. Additional trials should focus on panobinostat's ability to resensitize multiple myeloma patients refractory/relapsed to second-generation IMIDs, newer proteasome inhibitors, and monoclonal antibodies, besides its ability to augment their therapeutic efficacy [87], as reported with other HDAC inhibitors [107–109]. Moreover, these encouraging results should warrant further confirmation in larger clinical trials. Overall, drug toxicity profile and unique mechanism of action render panobinostat rather a more suitable candidate for multidrug regimens in heavily pretreated patients with aggressive disease behavior than in untreated patients or in first relapse. Ongoing trials (Table 2) are going to shed light on the optimal timing, schedule, and combinations of panobinostat in order to attain the maximum benefit from this class of anticancer agents and optimize treatment tolerability.

## 9. Conclusion

Despite significant advances in the therapeutic management of multiple myeloma, panobinostat remains an important therapeutic option for refractory/relapsed multiple myeloma patients. Available data from multiple clinical trials confirm that it remains an effective therapy even in heavily pretreated patients. HDAC inhibitors like panobinostat, as a part of multidrug combinations, may reform the current multiple myeloma treatment landscape allowing patients to achieve improved quality of response and prolonged survival, with affordable toxicity.

## Figures and Tables

**Table 1:** Drug summary.

Generic drug name	Panobinostat

Market name (pharmaceutical company)	Farydac® (Novartis)

Phase	Approved

Indication	In combination with bortezomib and dexamethasone, for the treatment of adult patients with relapsed and/or refractory multiple myeloma who have received at least two prior regimens including bortezomib and an immunomodulatory agent

Pharmacology description/mechanism of action	HDAC inhibitor

Route of administration	Oral

Chemical structure	2-Hydroxypropanoic acid, compound with 2-(E)-*N*-hydroxy-3-[4-[[[2-(2-methyl-1H-indol-3-yl)ethyl]amino]methyl]phenyl]-2-propenamide [1 : 1]

Pivotal trial (s)	Panorama 1

Metabolism and elimination	Extensively metabolized, eliminated equally through the kidney and liver

Main toxicities	Thrombocytopenia, anemia, neutropenia, diarrhea, fatigue

**Table 2:** Summary of all ongoing active trials investigating panobinostat in patients with MM.

A/a	NCT number (study name)	Phase	Comparing arms	Intervention/treatment	Patients characteristics
1	NCT01496118	I/II	Experimental: carfilzomib and panobinostat	Carfilzomib and panobinostat	Relapse/refractory after at least one previous bortezomib-containing regimen

2	NCT01965353	I	Experimental: panobinostat, dexamethasone, lenalidomide, and bortezomib	Dexamethasone and lenalidomide and bortezomib and panobinostat	Relapse/refractory after at least two lines of treatment

3	NCT02722941	II	Panobinostat: 20 mg by mouth three [3] times per week, every other week, of a 28-day schedule Panobinostat: 10 mg by mouth daily for seven [7] days, every other week, of a 28-day schedule	Panobinostat as maintenance therapy after HDT + ASCT	Patients must have achieved at least PR after ASCT

4	NCT03256045	II	Experimental: carfilzomib and panobinostat	Carfilzomib and panobinostat	Relapse/refractory after at least one previous line of treatment

5	NCT01301807	I	Experimental: carfilzomib and panobinostat	Carfilzomib and panobinostat	Relapsed/refractory MM with failure to at least two lines of MM treatment including at least one IMiD (thalidomide, lenalidomide) and proteasome inhibitor (bortezomib)

6	NCT02057640	I/II	Experimental: ixazomib, panobinostat, and dexamethasone	Ixazomib and panobinostat and dexamethasone	Relapsed/refractory MM after receiving at least one IMiD (thalidomide, lenalidomide) and proteasome inhibitor (bortezomib)

7	NCT00918333	I/II	Experimental: everolimus and panobinostat	Everolimus and panobinostat	Relapsed/refractory myeloma requiring therapy, who have failed, are unable to tolerate, or refused other available active therapies

8	NCT02506959	II	Experimental: panobinostat, gemcitabine hydrochloride, busulfan, and melphalan ASCT	Panobinostat, Gemcitabine Hydrochloride, Busulfan, and melphalan ASCT	Refractory/relapsed myeloma

9	NCT02654990	II	Experimental: panobinostat, bortezomib, and dexamethasone Investigate 3 different panobinostat regimens	Panobinostat and bortezomib and dexamethasone	Refractory/relapsed myeloma with 1 to 4 prior lines of treatment
